# Association between cardiorespiratory fitness and the prevalence of metabolic syndrome among Korean adults: a cross sectional study

**DOI:** 10.1186/1471-2458-14-481

**Published:** 2014-05-21

**Authors:** Sunghyun Hong, Junga Lee, Jihye Park, Mikyung Lee, Ji Young Kim, Kyong-Chol Kim, Sun Hyun Kim, Jee Aee Im, Sang Hui Chu, Sang Hoon Suh, Sang Hwan Kim, Justin Y Jeon

**Affiliations:** 1 Department of Sport and Leisure Studies, Sport Medicine Laboratory, Yonsei University, Seoul, Korea; 2 Exercise Medicine Center for Diabetes and Cancer Patients, Yonsei University, Seoul, Korea; 3 Anti-aging Center, Chaum Life Center, Cha Universty, Seoul, Korea; 4 Department of Family Medicine, Kwandong University, College of Medicine, Gangneung-si, Republic of Korea; 5 Sport and Medicine Research Center, INTOTO Inc, Seoul, Korea; 6 Department of Clinical Nursing Science, Nursing Policy Research Institute, Yonsei University College of Nursing, Seoul, Korea; 7 Department of Physical Education, Yonsei University, Seoul, Korea; 8 Institute of Geriatric Medicine, Yonsei Woori Geriatric Hospital, Goyang, Gyeonggi-do, Korea

**Keywords:** Cardiorespiratory fitness, Simple step test, Obesity, Metabolic syndrome, Korean adults

## Abstract

**Background:**

The purpose of the current study was to investigate the association between cardiorespiratory fitness (CRF), measured by a simple step test, and the prevalence of metabolic syndrome among Korean adults, in a cross sectional design.

**Methods:**

A total of 1,007 Korean adults (488 men and 519 women) who underwent routine health checkups were recruited. CRF was measured by Tecumseh step test. The National Cholesterol Education Program’s Adult Treatment Panel III guideline was used to determine the prevalence of metabolic syndrome. A logistic regression was performed to reveal possible associations.

**Results:**

The results of the study showed that a lower level of CRF was significantly associated with a higher prevalence of metabolic syndrome in men, but not in women. On the other hand, higher BMI was associated with a higher prevalence of metabolic syndrome in both men and women. However, BMI was not associated with fasting glucose nor hemoglobinA1c in men. When the combined impact of BMI and CRF on the prevalence of metabolic syndrome was analyzed, a significantly increased prevalence of metabolic syndrome was found in both men (odds ratio [OR]: 18.8, 95% Confidence Interval [CI]: 5.0 - 70.5) and women (OR: 8.1, 95% CI: 2.8 - 23.9) who had high BMI and low cardiorespiratory fitness. On the other hand, the prevalence of metabolic syndrome was only increased 7.9 times (95% CI: 2.0 - 31.2) in men and 5.4 times (95% CI: 1.9 - 15.9) in women who had high level of CRF and high BMI.

**Conclusion:**

In conclusion, the current study demonstrated the low CRF and obesity was a predictor for metabolic syndrome in Korean adults.

## Background

Metabolic syndrome, also known as insulin resistance syndrome or syndrome X, is the term most commonly used to identify a constellation of metabolic disorders [1]. In 2001, the National Cholesterol Education Program’s Adult Treatment Panel III (NCEP: ATP III) defined metabolic syndrome as having three of the five following components: glucose intolerance, hypertension, insulin resistance, abdominal obesity, and dyslipidemia [2]. Metabolic syndrome is associated with an increased risk of type 2 diabetes [3-5] and cardiovascular disease (CVD) [4,6].

Over the last few decades, the prevalence of metabolic syndrome has increased worldwide, and it has become a major public health concern in many countries, including several Asian countries [7-9]. According to data from the Korean National Health and Nutrition Examination Survey (KNHANES, 1998–2007), the age-adjusted prevalence of metabolic syndrome among Korean adults rapidly increased from 24.9% in 1998 to 31.3% in 2007 [9]. The rise in the prevalence of metabolic syndrome was related to both an increased prevalence of obesity [10,11] and decreased physical activity levels [12-14].

A low level of cardiorespiratory fitness (CRF) is a major contributing risk factor for metabolic syndrome [15-17], as well as a predictor of overall mortality [18-22]. Other studies showed that a high level of CRF is associated with a significantly reduced incidence of metabolic syndrome [15,23-25]. Thus, maintaining and improving CRF is an important strategy for the prevention of metabolic syndrome.

While several studies have indicated that increased CRF and decreased BMI reduced the risk of metabolic syndrome [15,24] the current study is one of the first studies to explore the combined association of BMI and CRF with the prevalence of metabolic syndrome in Korean adults. Most previous studies have used maximal oxygen consumption (VO2 max) to measure CRF, which has been the gold standard measurement for CRF [26]. However, this method is challenging due to the high cost of the equipment required and the need for trained personnel. For this reason, heart rate during or after submaximal exercise has been used as an alternative method to measure CRF in large epidemiological studies [24,27,28]. Furthermore, the step test has been proven to be safe and inexpensive, and therefore can be used in the clinic for patients with a history of cardiac events, as well as in situations where the equipment needed to perform the VO2 max test is not available.

Therefore, the purpose of this study was to analyze the association between CRF, measured by heart rate recovery (HRR) after the step test, and the prevalence of metabolic syndrome in Korean adults. We further analyzed the combined association of BMI and CRF with the prevalence of metabolic syndrome.

## Methods

### Ethical considerations

The study was approved by the Ethics Review Committee of MizMedi Hospital.

### Participants

The study recruited 1,007 Korean adults (488 men and 518 women) who visited the Health Care Center at Myongji University Hospital for general health examination between November 2008 and February 2009. The purpose of this study was explained to all participants, and written consent was obtained from all participants prior to their participation. Potential participants were excluded if they had experienced excessive body weight gain or loss (approximately ± 5 kg) during the past three months, or if they required additional medical monitoring due to cardiac or cancer-associated surgery during the past three months.

### Anthropometric and biochemical data

#### Anthropometric measurements

Height and weight were measured to the nearest 0.1 cm and 0.1 kg (JENIX DS-102; DONG SAHN JENIX Co Ltd, Seoul, Korea), with the participants barefoot and in light clothing. Body mass index (BMI) was calculated as weight (kilograms) divided by height (meters squared). Waist circumference (WC) was measured at the midpoint between the bottom of the rib cage and the top of the lateral border of the iliac crest with participants in the standing position at the end of a normal expiration. Blood pressure was measured twice at a five-minute interval. Blood pressure readings were taken from the right arm, after a rest period, by use of a sphygmomanometer (HM-1101; HICO Co Ltd, Tokyo, Japan).

#### Blood specimens

Blood samples were collected in the morning after participants had been seated for 30 minutes and had fasted overnight (at least 12 hours). Serum fasting glucose, total cholesterol (TC), triglycerides (TG), and high-density lipoprotein (HDL) were measured using an ADVIA 1650 Chemistry Analyzer System (Siemens, Tarrytown, NY, USA). Hemoglobin A1c (HbA1c) was evaluated using an HLC-723GHb (TOSOH, Siba, Japan).

### Definition of metabolic syndrome

This study used the National Cholesterol Education Program Adult Treatment Panel III (NCEP-ATP III) guidelines [2] to determine the presence of metabolic syndrome. Abdominal obesity was determined by waist circumference. To address ethnic and regional factors in the diagnostic criteria, abdominal obesity was defined by the Asia-Pacific criteria for waist circumference (APC-WC) [29]. The study used the recent International Diabetes Federation (IDF) metabolic syndrome definition [30], which includes criteria established by the NCEP: ATP III and the APC-WC. Participants were considered to have metabolic syndrome if three or more of the following five criteria were met: 1) high blood pressure (≥130/85 mmHg); 2) hyperglycemia (fasting plasma glucose ≥ 100 mg/dL); 3) hypertriglyceridemia (≥150 mg/dL); 4) low HDL cholesterol (<40 mg/dL in men, < 50 mg/dL in women); and 5) abdominal obesity (waist circumference ≥ 90 cm for men, ≥ 80 cm for women).

### Assessment of CRF

All participants underwent the Tecumseh step test to determine their CRF values. The test was performed on a step/bench 20.3 cm (8 inches) in height, for a duration of three minutes at a 24-cycle per minute rate, as controlled by a metronome [31]. One stepping cycle consisted of four steps: right foot UP, left foot UP, right foot DOWN, and left foot DOWN. Immediately following the three minutes of exercise, participants rested in a sitting position. The test procedure was demonstrated and explained to the participant before the onset of the exercise. Participants wore a heart rate monitor (Polar-FS3c, USA). Resting heart rates were measured prior to the test and at one-minute intervals during exercise, and then again one minute after termination of the exercise. The study defined HRR as the heart rate (beats/min) measured one minute after exercise. For analysis purposes, the participants’ data were separated on the basis of gender and categorized into one of three groups according to CRF tertile.

### Statistics

From the distribution of measurements calculated for body mass and fitness levels, the BMI and CRF values were divided into low, middle, and high groupings [32,33]. In men, the mean of low, middle, and high tertiles for BMI and CRF represented 21.7 (range 17.1 to 23.6), 24.7 (23.7 to 25.7), 27.4 (25.8 to 34) kg/m^2^ and 74.7 (53 to 82), 87.6 (83 to 93), 104.5 (94 to 137) beats/min, respectively; in women, the low, middle, and high tertiles for BMI and CRF represented 20.1 (15.9 to 21.4), 22.7 (21.5 to 24), 26.7 (24.1 to 35.6) kg/m^2^ and 79.8 (65 to 86), 92.4 (87 to 97), 108.2 (98 to 133) beats/min, respectively. Overall, participants were divided into nine groups: (1) low BMI and low CRF, (2) low BMI and middle CRF, (3) low BMI and high CRF, (4) middle BMI and low CRF, (5) middle BMI and middle CRF, (6) middle BMI and high CRF, (7) high BMI and low CRF, (8) high BMI and middle CRF, and (9) high BMI and high CRF, to investigate the relationships between metabolic syndrome, BMI and CRF.

The normality of the data was tested using the Shapiro-Wilk test. The parameters’ normality was compared with the Student’s t-test, and the nonparametric Mann–Whitney test was conducted for non-normally distributed variables. Statistical comparisons among the tertile groups for HRR and BMI were performed using one-way analysis of variance (ANOVA) followed by a post-hoc Scheffe for men and analysis of covariance (ANCOVA) with post-hoc Bonferroni for women, respectively. To determine the association of metabolic syndrome prevalence with CRF and BMI, logistic regression analyses were performed after adjusting for age. Metabolic syndrome was assigned as a dependent variable, and age, HRR and BMI were assigned as independent variables. A two-sided analysis with *p* < 0.05 was considered statistically significant. All data are presented as mean ± standard deviation (SD) and percentages. All statistical analyses were conducted using SPSS version 18.0 for Windows.

## Results

### Clinical characteristics

The anthropometric and biochemical characteristics of participants in this study are summarized in Table 1. Of the 1,007 participants, 110 (22.9%) of the 488 men, and 119 (23.1%) of the 518 women had metabolic syndrome.

**Table 1:** Characteristics of the study participants (n = 1007)

	**Men (n = 488)**	**Women (n = 519)**	**Total (n = 1007)**	***p *** **Value**
Anthropometric				
Age (years)	51.11 ± 13.93	49.54 ± 13.51	50.30 ± 13.74	0.359
Height (cm)	170.18 ± 5.52	158.06 ± 5.70^#^	163.93 ± 8.26	0.761
Weight (kg)	71.54 ± 10.01	57.82 ± 8.25^#^	64.47 ± 11.43	0.038
BMI (kg/m^2^)	24.61 ± 2.69	23.17 ± 3.21^#^	23.87 ± 3.05	0.001
Waist circumference (cm)	84.44 ± 7.07	74.19 ± 8.11^#^	79.18 ± 9.18	0.004
Blood pressure				
Systolic blood pressure (mmHg)	125.08 ± 12.23	121.01 ± 14.80^#^	122.99 ± 13.76	0.001
Diastolic blood pressure (mmHg)	77.58 ± 8.82	72.18 ± 9.80^#^	74.80 ± 9.71	0.013
Blood variables				
Glucose (mg/dl)	104.33 ± 24.40	95.30 ± 14.66^#^	99.67 ± 20.47	<0.001
Hemoglobin A1C (mg/dl)	5.67 ± 0.71	5.67 ± 0.75	5.67 ± 0.73	0.46
Total cholesterol (mg/dl)	192.47 ± 33.12	190.06 ± 33.14	191.23 ± 33.13	0.647
Triglycerides (mg/dl)	153.27 ± 95.90	109.44 ± 67.18^#^	130.49 ± 85.06	<0.001
Low-density lipoprotein (mg/dl)	119.79 ± 29.73	120.44 ± 30.65	120.13 ± 30.20	0.185
High-density lipoprotein (mg/dl)	45.66 ± 10.80	53.53 ± 12.25^#^	49.75 ± 12.22	0.008
Number of metabolic syndrome components				
0	105 (21.5)	49 (9.4)	154 (15.3)	
1	146 (29.9)	198 (38.2)	344 (34.2)	
2	117 (24.0)	149 (28.7)	266 (26.4)	
3	71 (14.5)	84 (16.2)	155 (15.4)	
4	24 (4.9)	25 (4.8)	49 (4.9)	
5	13 (2.7)	5 (1.0)	18 (1.8)	
Metabolic syndrome	110 (22.9)	119 (23.1)	229 (22.7)	

Data are presented as means ± SD or *N* (%).

*p* values for categorical variables were determined from the Student’s t-test for continuous data.

Number of MS components was not equal to actual number of participants with metabolic syndrome due to missing data (men: 12; women: 9).

^*^p < 0.05 vs. men, ^#^*p* < 0.01 vs. men.

### Association between CRF and metabolic syndrome

Anthropometric and metabolic parameters according to tertile of HRR after step exercise are presented in Table 2. There was no difference in any of the anthropometric components among the groups. The fasting glucose level was significantly higher and the high-density lipoprotein (HDL-C) was significantly lower in the low CRF group compared with those in the moderate and high CRF groups (*p* < 0.05). The low CRF group had a significantly higher number of participants with metabolic syndrome when compared to the high CRF group in men, but the same was not true in women.

**Table 2:** Anthropometric and exercise characteristics of men (488) and women (519) according to tertile of BMI

	**Men (n = 488)**				**Women (n = 519)**			
	**Low BMI (17.1 – 23.6 kg/m**^**2**^**)**	**Middle BMI (23.7 – 25.7 kg/m**^**2**^**)**	**High BMI (25.8 - 34 kg/m**^**2**^**)**	***p *** **Value**	**Low BMI (15.9 – 21.4 kg/m**^**2**^**)**	**Middle BMI (21.5 – 24 kg/m**^**2**^**)**	**High BMI (24.1 – 35.6 kg/m**^**2**^**)**	***p *** **Value**
No. of participants	162	166	160		169	179	171	
Anthropometric								
Age (years)	51.06 ± 15.49	51.02 ± 13.34	51.24 ± 12.94	0.989	44.24 ± 12.90	49.21 ± 13.23	55.13 ± 12.22	< 0.001
Height (cm)	170.48 ± 5.41	170.25 ± 5.66	169.79 ± 5.50	0.524	158.70 ± 5.27	157.74 ± 5.12	157.97 ± 5.29	0.215
Weight (kg)	63.13 ± 6.23	71.76 ± 4.66^*^	79.82 ± 10.19^*#^	< 0.001	50.41 ± 5.47	56.50 ± 5.32^*^	66.64 ± 5.49^*#^	< 0.001
BMI (kg/m^2^)	21.69 ± 1.54	24.74 ± 0.60^*^	27.44 ± 1.61^*#^	< 0.001	20.05 ± 1.65	22.69 ± 1.61^*^	26.73 ± 1.67^*#^	< 0.001
Waist circumference (cm)	77.84 ± 5.29	85.07 ± 3.99^*^	90.48 ± 5.16^*#^	< 0.001	67.70 ± 4.77	72.89 ± 4.64^*^	81.99 ± 4.78^*#^	< 0.001
Blood pressure								
Systolic blood pressure (mmHg)	121.48 ± 13.04	124.95 ± 10.44	128.87 ± 12.03^*#^	< 0.001	116.78 ± 12.94	119.85 ± 12.57	125.75 ± 12.99^*#^	< 0.001
Diastolic blood pressure (mmHg)	74.75 ± 9.18	77.90 ± 8.07	80.10 ± 8.41^*#^	< 0.001	70.09 ± 9.32	71.74 ± 9.06	74.38 ± 9.35^*#^	< 0.001
Blood variables								
Glucose (mg/dl)	103.98 ± 31.10	102.96 ± 20.94	106.09 ± 19.58	0.502	92.07 ± 14.66	96.34 ± 14.25^*^	97.38 ± 4.71^*^	0.003
Hemoglobin A1C (mg/dl)	5.67 ± 0.81	5.68 ± 0.63	5.67 ± 0.68	0.990	5.70 ± 0.76	5.76 ± 0.75	5.55 ± 0.78^#^	0.034
Total cholesterol (mg/dl)	192.21 ± 23.17	194.51 ± 34.51	190.60 ± 32.68	0.563	190.79 ± 34.22	190.01 ± 33.27	189.76 ± 34.34	0.961
Triglycerides (mg/dl)	127.91 ± 86.02	145.01 ± 75.52	187.15 ± 133.32^*^	< 0.001	96.49 ± 64.19	104.67 ± 62.43	124.59 ± 64.42^*#^	< 0.001
Low-density lipoprotein (mg/dl)	118.96 ± 28.75	122.89 ± 31.55	117.34 ± 28.62	0.230	120.15 ± 31.59	122.40 ± 30.72	119.02 ± 31.70	0.586
High-density lipoprotein (mg/dl)	49.15 ± 12.33	45.19 ± 10.60^*^	42.65 ± 8.11^*#^	< 0.001	57.43 ± 12.20	53.26 ± 11.87^*^	50.24 ± 12.24^*^	< 0.001
No.of participants with MS	14 (8.9)	30 (18.4)	66 (41.5)^*#^	< 0.001	17 (10.1)	31 (17.6)	71 (41.5)^*#^	< 0.001
Odd ratio (95% confidence interval)	1 (reference)	2.48 (1.24-4.96)	8.23 (4.27-15.85)		1 (reference)	1.63 (0.86-3.11)	4.64 (2.53-8.53)	

Data are presented as means ± SD or *N* (%).

All men and women participants were divided into three groups according to tertile of Body Mass Index, respectively.

One-way ANOVA was used to compare means of parameters between three groups in men. Post-hoc analysis: Scheffe.

Analysis of Covariance (ANCOVA) was used to compare means of parameters between three groups after age controlled for women. Post-hoc analysis: Bonferroni.

^*^significantly different between low BMI and middle BMI or significantly different between low BMI and high BMI, *p* < 0.05, 1^#^significantly different between middle BMI and high BMI, *p* < 0.05.

*Abbreviations*: *BMI* Body Mass Index, *CRF* Cardiorespiratory fitness.

### Association between adiposity and metabolic syndrome

Anthropometric and metabolic parameters according to tertile of BMI are shown in Table 3. When participants were stratified into tertile based on their BMI, the levels of fasting glucose and HbA1c were found to be associated with BMI in women, but not in men. In general, the more obese participants had worse lipid profiles in both the men and women categories. The high BMI group had significantly higher triglycerides and a significantly lower HDL-C. The high BMI group had a significantly higher number of participants with metabolic syndrome when compared to the low BMI group in men and in women.

**Table 3:** Anthropometric and exercise characteristics of men (488) and women (519) according to tertile of heart rate recovery

	**Men (n = 488)**				**Women (n = 519)**			
	**High CRF (53 – 82 bpm)**	**Moderate CRF (83 – 93 bpm)**	**Low CRF (94 – 137 bpm)**	***p *** **Value**	**High CRF (65 – 86 bpm)**	**Moderate CRF (87 – 97 bpm)**	**Low CRF (98 – 133 bpm)**	***p *** **Value**
No. of participants	163	162	163		176	169	174	
Anthropometric								
Age (years)	52.56 ± 14.04	51.49 ± 13.70	49.26 ± 13.95	0.092	54.74 ± 12.95	48.31 ± 13.45^*^	45.48 ± 12.46^*^	< 0.001
Height (cm)	169.62 ± 5.50	170.16 ± 5.50	170.75 ± 5.54	0.182	158.15 ± 8.54	158.14 ± 5.17	158.11 ± 8.90	1.000
Weight (kg)	70.29 ± 8.54	72.53 ± 11.82	71.54 ± 9.31	0.121	56.42 ± 13.68	57.86 ± 8.29	59.18 ± 14.27	0.377
BMI (kg/m^2^)	24.41 ± 2.50	24.82 ± 2.70	24.61 ± 2.85	0.391	22.62 ± 5.05	23.15 ± 3.06	23.62 ± 5.26	0.362
Waist circumference (cm)	83.45 ± 6.80	84.90 ± 7.02	84.97 ± 7.31	0.089	72.71 ± 12.02	74.08 ± 7.28	75.58 ± 12.52	0.242
Blood pressure								
Systolic blood pressure (mmHg)	125.18 ± 12.38	124.07 ± 11.46	125.99 ± 12.80	0.368	119.69 ± 21.63	119.24 ± 13.13	122.73 ± 22.56	0.347
Diastolic blood pressure (mmHg)	76.50 ± 8.93	77.19 ± 8.44	79.05 ± 8.93^*^	0.026	72.28 ± 15.25	71.13 ± 9.26	73.74 ± 15.91	0.287
Blood variables								
Glucose (mg/dl)	100.40 ± 16.53	102.20 ± 20.95	110.36 ± 31.99^*#^	< 0.001	94.17 ± 23.94	94.65 ± 14.51	96.95 ± 24.97	0.601
Hemoglobin A1C (mg/dl)	5.67 ± 0.68	5.66 ± 0.75	5.68 ± 0.70	0.982	5.68 ± 1.25	5.61 ± 0.75	5.71 ± 1.29	0.409
Total cholesterol (mg/dl)	192.33 ± 33.54	192.99 ± 32.85	192.08 ± 33.16	0.968	182.75 ± 55.09	192.22 ± 33.40	195.47 ± 57.47.	0.136
Triglycerides (mg/dl)	140.04 ± 85.72	162.97 ± 109.63	158.89 ± 89.58	0.086	103.56 ± 105.30	113.94 ± 63.74	107.88 ± 109.82	0.402
Low-density lipoprotein (mg/dl)	120.46 ± 29.70	117.53 ± 30.09	121.36 ± 29.47	0.492	113.03 ± 50.81	123.42 ± 30.81^*^	125.15 ± 53.01	0.061
High-density lipoprotein (mg/dl)	48.23 ± 13.02	44.25 ± 8.93^*^	44.47 ± 9.58^*^	0.001	54.47 ± 20.27	53.67 ± 12.27	52.83 ± 21.14	0.851
No. of participants with MS	26 (23.6)	39 (35.5)	45 (40.9)^*^	0.035	39 (22.4)	37 (21.9)	43 (24.9)	0.392
Odd ratio (95% confidence interval)	1 (reference)	1.78 (1.01-3.15)	2.40 (1.37-4.22)		1 (reference)	1.36 (0.79-2.34)	1.99 (1.12-3.34)	

Data are presented as means ± SD or *N* (%).

All men and women participants were divided into three groups according to tertile of Heart Rate Recovery, respectively.

One-way ANOVA was used to compare means of parameters between three groups in men. Post-hoc analysis: Scheffe.

Analysis of Covariance (ANCOVA) was used to compare means of parameters between three groups of women after age was controlled for. Post-hoc analysis: Bonferroni.

^*^significantly different between low CRF and middle CRF or significantly different between low CRF and high CRF; ^#^significantly different between middle CRH and high CRF.

*Abbreviations*: *HRR* Heart rate recovery, *CRF* Cardiorespiratory fitness.

### Combined impact of BMI and CRF on the prevalence of metabolic syndrome

**Figure 1:**
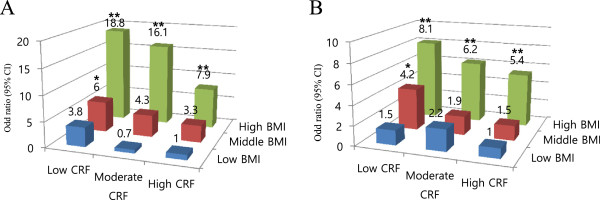
**Adjusted Odd ratio (95% CI) for metabolic syndrome according to cardiorespiratory fitness (CRF) and body mass index (BMI). (A)** Men. **(B)** Women. Adjusted for age. Statistically significant different from reference group: *p < .05, **p < .01. The participants were divided into nine groups according to tertile of their CRF and BMI. For BMI, the tertile ranges in men were: low BMI, 17.1-23.6; middle BMI, 23.7-25.7; high BMI, 25.8-34 kg/m^2^; and in women: low BMI, 15.9-21.4; middle BMI, 21.5-24; high BMI, 24.1-35.6 kg/m^2^_._ For CRF, the tertile range in men: low CRF, 94-137; moderate CRF, 83-93; high CRF, 53-82 beats/min; and in women: low CRF, 98-133; moderate CRF, 87-97; high CRF, 65-86 beat/min.

To investigate the combined association of BMI and CRF with the prevalence of metabolic syndrome, the participants were stratified into nine subgroups, according to their BMI and CRF tertile (Figure 1).

Participants in the high BMI and low CRF group had 18.8 times (95% CI: 5.0 - 70.5) and 8.1 times (95% CI: 2.8 - 23.9) higher prevalence of metabolic syndrome in men and women, respectively. The prevalence of metabolic syndrome was reduced among participants in the high-BMI group if their CRF level was also high (OR: 7.9, 95% CI: 2.0-31.2 for men, OR: 5.4, 95% CI: 1.9-15.9 for women).

## Discussion

Reductions in physical activity and CRF are associated with increased prevalence and incidence of metabolic syndrome. In our study, we found that a lower level of CRF, as indicated by slower HRR following exercise, was associated with increased prevalence of metabolic syndrome in Korean men. A low level of CRF is a known risk factor for both cardiovascular disease and type 2 diabetes; however, the importance of the current study is the use of HRR after submaximal exercise as a measure of CRF. A prior study has validated the Tecumseh step test as an appropriate measurement to indicate cardiorespiratory fitness [34]. In comparison to the other more elaborate and expensive test approaches previously used to obtain VO2 max, the Tecumseh step test, used in the present study, is a relatively quick and easy method that can be used in most epidemiological and clinical settings [34]. Findings from the current study also indicate that the association between CRF and the prevalence of metabolic syndrome was somewhat gender dependent, although this relationship was less clear when the combined association of BMI and CRF with metabolic syndrome prevalence was examined.

The main finding of this study is the significant association between faster HRR after submaximal exercise and the lower prevalence of metabolic syndrome in men, suggesting that fitter men participants are at lower risk of metabolic syndrome. The association between CRF and metabolic syndrome has been reported previously. Laaksonen et al. [17] reported a significant inverse association between CRF and prevalence of metabolic syndrome even after adjustment for major confounders. In addition, Lamonte et al. [15] reported that the incidence of metabolic syndrome was significantly reduced among fit individuals (OR: 0.47, 95% CI: 0.40 to 0.54) compared with the least fit individuals (lower tertile). The current study and previously reported studies suggest that fitter individuals are less likely to develop metabolic syndrome compared with those who are unfit. However, physical fitness is not the only contributor for the development of metabolic syndrome.

There are other factors independent of CRF that influence the development of metabolic syndrome. In our study, approximately 50% of obese individuals had metabolic syndrome. Similarly, several previous studies found that the components of metabolic syndrome were closely associated with obesity [10]. In a prospective cohort study, Katzmarzyk et al. [35] reported that overweight men were 4.5 times (95% CI: 4.2-5.3) more likely to develop metabolic syndrome, and obese men were 30.6 times (95% CI: 26.7-35.0) more likely to develop metabolic syndrome. It is not surprising that more obese individuals have a higher prevalence of metabolic syndrome; one of the five metabolic syndrome components directly reflects degree of adiposity. In our study, we also confirmed that more obese individuals are more likely to have metabolic syndrome.

To further understand the combined association of obesity and CRF with the prevalence of metabolic syndrome, we have stratified our participants into nine groups according to their BMI and fitness levels. Compared with those who had low BMI and high CRF, those with high BMI and low CRF were 18.8 and 8.1 times more likely to have metabolic syndrome in men and women, respectively. It is noteworthy that among those with high BMI, fitter individuals of either gender have a lower risk of metabolic syndrome, suggesting the importance of fitness in the development of metabolic syndrome in Korean adults. For this study, CRF was indicated by the HRR measurement taken after administering the Tecumseh step test. Previous epidemiological studies have typically used the graded treadmill test or the VO2 max to measure CRF, when investigating the association between CRF and health-related outcomes. However, administering the VO_2 max_ test requires specialized and expensive equipment, in addition to requiring well-trained technicians [34]. Recent studies indicate that CRF can more conveniently be assessed by measuring HRR after treadmill exercise. This HRR approach combined with treadmill exercise is a safe and inexpensive method, and has been proven to be valid [34-36]. An additional benefit from using the HRR approach lies in its demonstrated value in treating key components of metabolic syndrome, to include positive outcomes for fasting plasma glucose [37], HDL cholesterol [38-40], and insulin levels [41], and prevalence of type 2 diabetes mellitus [42]. Therefore, based on the findings from these studies [37-42], and supported by previously reported studies, we conclude that HRR following exercise is a safe and feasible method for estimating CRF in an epidemiological setting [28].

In our study, we have observed gender differences in the association of BMI and CRF with the components of metabolic syndrome. CRF was significantly associated with fasting glucose and HbA1c in men only, while BMI was significantly associated with fasting glucose and HbA1c in women only. The discrepancies found between genders could be due to the gender-specific distribution of adiposity and level of physical activity. Kriska et al. [36] reported that physical activity and physical fitness were associated with fasting glucose levels in men, but not in women. Authors speculated the reason for this gender difference was due to lower levels of physical activity in women participants. Indeed, in our previously reported study with the same cohort, we found significantly lower vigorous, moderate, and total physical activity levels in women participants compared with men [14]. Another explanation for the gender differences observed in our study could be the distribution of adiposity. Waist circumference and BMI were significantly lower among women participants compared with men participants in our study. Meanwhile, our women participants with high BMI still had significantly increased metabolic risk factors. The gender differences observed in our study require further investigation.

In understanding the mechanism of association between HRR and the prevalence of metabolic syndrome, independent and dependent of adiposity, it is necessary to understand the impact of parasympathetic and sympathetic nervous system activity on heart rate. The changes in cardiovascular function associated with low-intensity exercise are primarily mediated by parasympathetic withdrawal, and, as exercise intensity increases, additional cardiovascular reactivity is mediated by increased sympathetic outflow [37]. The fall in heart rate immediately after exercise is considered to be a function of the reactivation of the parasympathetic nervous system [38]. Delayed HRR after exercise could be related to attenuated parasympathetic reactivity, which has been described as a marker of decreased vagal activity [39]. Attenuated HRR induced by decreased vagal activity is a powerful risk factor for all-cause mortality [39-43].

The current study has several limitations. First, the level of HRR from the Tecumseh step test might be affected by BMI. The high BMI group could have an increase in heart rate that is greater than those of the other groups when they step up and down. Despite this limitation, this step test has been frequently used in clinical settings as a representative CRF test [44-46]. Second, due to the cross-sectional nature of this study, it was not possible to control some confounding factors as their diet and medications that may have affected the results. Factors that could have produced confounding influences included that the participants were recruited in this study by using convenience sampling, a relatively small sample size was used, and the limited age range for the group (middle age). Because of these biases and limitations, it is difficult to maintain that the findings of the present study accurately represent the Korean population in general.

## Conclusions

In conclusion, we found that participants with a high level of CRF have lower risk of metabolic syndrome. The prevalence of metabolic syndrome increased as the degree of adiposity increased. However, high levels of CRF were associated with lower prevalence of metabolic syndrome among obese individuals. Our findings suggest the importance of physical fitness in the prevention of metabolic syndrome.

## Competing interests

The authors declare that they have no competing interests.

## Authors’ contributions

JJ conceived and designed the study. HSH and LJA conducted the data analyses and drafted the manuscript. JJ led the drafting of manuscript and prepared the final version. All authors revised the manuscript for important content and have read and approved the final version.

## Pre-publication history

The pre-publication history for this paper can be accessed here:

http://www.biomedcentral.com/1471-2458/14/481/prepub
